# Concurrent thrombotic thrombocytopenic purpura and Guillian Barre Syndrome post infectious diarrhea

**DOI:** 10.1016/j.amsu.2022.104301

**Published:** 2022-08-05

**Authors:** Taimoor Hussain, Jasvindar Kumar, Sarab Jeet Singh, Anil Kumar, Ihtisham Qayum

**Affiliations:** a Bolan Medical College Quetta, Pakistan; b Khyber Teaching Hospital Peshawar, Pakistan; c Federal Govt. Polyclinic Hospital Islamabad, Pakistan; d Saidu Group of Teaching Hospital, Pakistan; e Russel Hall Hospital Dudley West Midlands, UK

**Keywords:** TTP, GBS, Concurrent thrombotic thrombocytopenic purpura and Guillian Barre syndrome, Molecular mimicry and cross reactivity

## Abstract

Thrombotic thrombocytopenic purpura (TTP) characterized by microangiopathic hemolytic anemia, thrombocytopenia and signs of organ dysfunction, is due to either congenital or acquired deficiency of ADAMTS13 gene. Guillian Barre Syndrome (GBS) is a post infectious disorder, most commonly associated with C. jejuni infection. Both conditions have high mortality if untreated and have been reported with other comorbid conditions. We found only one case report of sequential TTP and GBS. However, we report the first case of concurrent TTP and GBS infection in a 22 years old female after bloody diarrhea, successfully managed by symptomatic treatment, sessions of plasmapheresis, and hemodialysis. TTP and GBS have both been associated with bacterial and viral infections, and antibodies formed against them may result in cross reactivity due to molecular mimicry. It is suggested although unproven that in such cases, patients likely developed cross-reactivity against both platelet and neurogenic glycoproteins (the linking antigen) following predisposing infection.

## Highlights

• TTP is due to deficiency of ADAMTS13, defined by microangiopathic hemolytic anemia, thrombocytopenia, and organ dysfunction.
• GBS is post-infectious disease, defined by progressive symmetric weakness of limbs, diminished or absent reflexes, and paresthesia.
• Both GBS and TTP have been reported with bacterial and viral infection including C. jejuni.
• In concurrent cases molecular mimicry and aberrant immune response is a plausible explanation.
• Such patients likely developed cross-reactivity against both platelet and neurogenic glycoproteins following an infection.

## 1 Introduction

Thrombotic thrombocytopenic purpura (TTP) is characterized by microangiopathic hemolytic anemia, thrombocytopenia and signs of organ dysfunction due to compromised microcirculation. TTP is a rare disorder with an average annual prevalence of ∼10 cases/million people and an annual incidence of ∼1 new case/million people [1,2]. ADAMTS13 is deficient in TTP, which can be caused either by genetic abnormalities (congenital TTP) or by autoantibodies affecting function or clearance of ADAMTS13 (autoimmune TTP). ADAMTS13 is a metalloprotease enzyme that cleaves von Willebrand factor (VWF) - a protein involved in blood clotting at the site of injury. Deficiency of ADAMTS13 results in uncleaved ultra large VWF multimeres (UL-VWF MM), with additional triggers results in enhanced platelet aggregation, resulting in organ damage due to compromised circulation [3]. TTP mainly affects the central nervous system, but the heart and kidneys are the other commonly affected organs.

Guillian Barre Syndrome (GBS) has a worldwide annual incidence of 1.1–1.8 per 100,000/year, that increases to 1.7–3.3 per 100,000/year in adults over 50 years [4]. GBS is characterized by a rapidly progressive bilateral, relatively symmetric weakness of the limbs, numbness, or paresthesia, with or without affecting respiratory muscles or cranial-nerve-innervated muscles [5]. In most of the patients GBS occurs after gastrointestinal or respiratory infections, where the antibodies formed against the bacteria cross react with the antigens on peripheral nerves due to molecular mimicry [5].

There are case reports of GBS or TTP associated with other diseases occurring concurrently or sequentially. We found only one case report of sequential TTP and GBS. However, this is the first case report describing concurrent TTP and GBS in a 22 years old female after bloody diarrhea. We successfully treated our patient with symptomatic treatment, 5 sessions of plasmapheresis, and 8 sessions of hemodialysis. The case is reported in line with the SCARE 2020 criteria [6].

## 2 Case presentation

According to the guardians of the patient, initially the 22 years old female had complaints of fever, vomiting and diarrhea. Fever was 101 ◦ F, intermittent and gradual in onset. She had complained of soft loose stools, 12 episodes per day, initial few episodes were mixed with blood. She also had complaints of vomiting. The vomitus contained food particles and 5–6 episodes per day. On 6th day of her illness the patient developed multiple small purple to reddish skin lesions on legs which then spread to the whole body. Her hospital course was further complicated by seizures and decreased power in lower limbs, and inability to walk by the end of second week of illness. Gradually in the third week of her illness she became disoriented, confused and could not respond to oral commands. She was initially being treated in district headquarter hospital but when her condition exacerbated, she was referred to tertiary care hospital. She did not give any history of allergies or medication use. She was non-smoker and did not use alcohol or drugs. Her past medical and surgical history were non-significant. Her family history was normal and did not report any genetic diseases. She had normal menstrual cycles with no history of abortion.

On general physical examination she had blood pressure of 100/60 mmHg, pulse was 67 beats/min, and 101◦ F temperature. She had pale looking skin and mild edema. Clubbing of nails or palpable lymphadenopathy was not observed. Her skin had small purplish to reddish skin lesions. The rashes were non blanchable, involved the whole body, ranging from 1 to 5 mm in size. Central nervous system examination revealed absent ankle reflexes, hypotonia and lower limbs power of 1/5 as per medical research council scale bilaterally, sensations were intact to soft touch and pinprick. Her vision was normal and had no present or past visual complains. Her Glasgow coma scale score was 6/15 (scoring 2 for each component of the scale). Babinski sign was negative bilaterally. Abdomen, respiratory and cardiovascular examinations were normal.

## 3 Investigations

Her initial lab tests on arrival in the tertiary care hospital revealed leukocytosis (38000/μL), anemia (Hb = 9.5 mg/dL), and thrombocytopenia (88000/μL). Her PT and INR were in normal range. APTT with Russel viper venom was reported normal. Peripheral blood smear showed shistocytes. Her serum Lactate dehydrogenase (LDH) was 2214 U/L and D-dimer was 900 ng/mL. Her serum urea and creatinine were 412 mg/dL and 12.6 mg/dL respectively. Her urine output was decreased. CSF analysis showed albuminocytologic disassociation (high protein and normal cell count). Serum ANA was negative. Blood, stool and urine culture showed no growth. Chest X-ray and ultrasound of kidneys, urinary bladder was reported normal. CT scan brain was reported normal which is usually performed rather than MRI because of being readily available, fast and more affordable. Nerve conduction studies (NCS) showed evidence of demyelinating peripheral radiculoneuropathy. The clinical presentation and NCS findings were suggestive of GBS. Pulse oxygen saturation was normal, and she did not have any signs of respiratory distress. Her guardians denied renal biopsy. In our set up we have financial and technological constraints, therefore auto antibodies to ADAMTS13 or gangliosides were not tested.

## 4 Differentials

A differential diagnosis of meningococcemia disseminated intravascular coagulation (DIC), TTP associated with concurrent GBS was made. Hemolytic uremic syndrome (HUS) was added to the list of differentials. Negative blood cultures, normal PT and PTT, fever and neurological signs ruled out meningococcemia, DIC, and HUS respectively. Our clinical and lab findings favored more the diagnosis of GBS and TTP post infectious diarrhea. The pentad of thrombocytopenia, fever, purpural skin lesions, altered mental status and deranged renal function supported the diagnosis of TTP. Uremic encephalopathy is a clinical diagnosis. It is possible that severe renal injury from TTP resulted in uremic encephalopathy and further aggravated the altered mental status. Albuminocytologic dissociation on CSF analysis, NCS result, absent reflexes and loss of power in lower limbs after bloody diarrhea favored GBS.

## 5 Treatment

She was shifted to intensive care unit and treated symptomatically with antipyretics, hydration and nutrition, intravenous broad-spectrum antibiotics, strict intake output record and anti-epileptics for seizures. Due to continuously deranged renal function tests, she was treated with 8 sessions of hemodialysis. Intravenous immunoglobulin (IVIg) is more expensive, requires strict monitoring and more beneficial if given in earlier weeks of illness, thus it was not preferred. Plasma exchange is more expensive. Patients and their attendants are non-affording and non-compliant when asked for required medications, fresh frozen plasma, and plasma exchange kits repetitively for the sessions. Our hospital has subsidized and affordable plasmapheresis facility. Therefore, she was given 5 sessions of plasmapheresis for GBS, to which she showed good response. Regular close monitoring of vitals, intake output record, daily complete blood count (CBC) and serum LDH level were advised.

LDH decreased from 2214 U/L to 370 U/L and platelets increased from 88,000/μL to 250,000/μL. After five sessions of plasmapheresis LDH and platelet count was rechecked for recurrence of disease. Her serum urea improved from 412 mg/dL to 112.6 mg/dL and creatinine decreased from 12.6 mg/dL to 5.03 mg/dL. Her urine output improved. She showed good compliance and did not develop any complications to the treatment. Power and reflexes were back to normal after treatment. She improved markedly and discharged with consultation of nephrologist. She was called for follow up in two weeks. On each subsequent follow up visits her renal function improved back to normal levels.

## 6 Clinical discussion

TTP is due to either congenital deficiency of ADAMTS13 gene or secondary to autoimmunity. Congenital ADAMTS 13 deficiency is due to mutations and polymorphisms in the ADAMTS13 gene [7]. Diagnosis is performed by demonstrating the lack of ADAMTS13 activity, ruling out *anti*-ADAMTS13 antibodies. Immune TTP is due to autoantibodies targeting ADAMTS13, resulting in either inhibition of function or enhanced clearance. Infections with viruses (Epstein–Barr virus, cytomegalovirus, HIV, etc.) or other pathogens, malignancy, certain drugs, other concomitant autoimmune diseases, pregnancy are known triggers of the autoimmunity, but in many cases cause remain unknown” [7]. Diagnosis is performed by demonstrating the absence of ADAMTS13 activity, and the detection of *anti*-ADAMTS13 antibodies [7].

As per an analysis of 14,400 cases of TTP approximately 9% had one or more underlying connective tissue disease [8]. TTP has been reported with systemic lupus erythematosus (SLE), Graves' disease, adult onset still's disease, membranous nephropathy, Good pasture disease, Legionella pneumonia, antiphospholipid antibody syndrome (APLA), multiple endocrine neoplasia 1 (MEN I) [[9], [10], [11], [12], [13], [14], [15], [16]].

On the other hand, in two thirds of patients GBS usually occurs after respiratory or gastrointestinal infection, 2–3 weeks prior to the onset of GBS symptoms and is not typically associated with an autoimmune or other systemic disorder [5]. The most frequently identified cause of infection is C. jejuni. Other well defined types of infection related to GBS are Cytomegalovirus, Epstein-Barr virus, Mycoplasma pneumoniae, and Haemophilus influenza [17,18]. There is strong evidence that GBS, at least in some cases, is the result of an infection-induced aberrant immune response damaging peripheral nerves.

GBS has been reported with other comorbid conditions like immune thrombocytopenic purpura (ITP). Sato et al. (2005) and Ian M Ward et al. (2013) reported a compilation of such cases in addition to their case of concurrent GBS and ITP [19,20]. GBS has also been reported with posterior reversible encephalopathy syndrome (PRES), Covid-19 infections, myasthenia gravis, nephrotic syndrome, pulmonary tuberculosis to name a few [[21], [22], [23], [24], [25]]. However, as per our review we found only one case reporting “leptospirosis complicated by sequential GBS, papillitis and TTP [26]. We are reporting the second case but the first one concurrently affected by GBS and TTP.

Campylobacter has been reported with TTP and GBS both [16,17,[26], [27]]. Occurrence of GBS is sometimes 77 times higher in those who had Campylobacter infection compared to the general population [28]. Serum antibodies to various gangliosides have been found in human peripheral nerves, in about half of patients with GBS. The carbohydrates of gangliosides such as GM1, GD1a, and GQ1b to name a few, resemble the lipo-oligosaccharides (LOS) of C. jejuni [[29], [30], [31]]. Though no bacterial or viral infections are known to directly lead to autoimmune TTP, molecular mimicry between ADAMTS13 and certain pathogens such as influenza A, *Helicobacter pylori*, Legionella, hepatitis C virus, and HIV may evoke an immune response [32]. As GBS and TTP both can be triggered by bacterial and viral infections resulting in cross reactivity due to molecular mimicry, their association may help in finding the linking antigen that predisposes to concurrent GBS and TTP. It is unproven though plausible that in such cases, patients likely developed cross-reactivity against both platelet and neurogenic glycoproteins post respiratory or gastrointestinal infectious disease.

Plasma exchange (PE) and Intravenous immunoglobulin (IVIg) are the mainstay treatment of GBS, especially in the first four weeks after onset of weakness [5]. According to a review on GBS, oral steroids or intravenous methylprednisolone alone are not beneficial in GBS. The combination of intravenous methylprednisolone and IVIg was not superior to IVIg alone. Similarly, the PE followed by IVIg is not significantly more effective than PE or IVIg alone [5].

PE therapy improves survival in TTP. Plasma infusion of ADAMTS13 is beneficial in congenital TTP [7]. Autoimmune types of TTP often respond to PE, immunosuppression and rituximab [7]. Future therapeutic options include; blocking the VWF-platelet interaction with *anti*-VWF A1 agents, replacement of ADAMTS13 by a recombinant ADAMTS13 concentrate, inhibition of VWF- Platelets interaction by Caplacizumab and *N*-acetyl cysteine [7]. Symptomatic treatment is provided for other complications as and when required based on the organ affected.

## 7 Conclusion

TTP and GBS can occur concurrently post infectious diarrhea. Both of them have both been associated with bacterial and viral infections, and antibodies formed against them may result in cross reactivity due to molecular mimicry. It is reasonable although unproven that in such cases, patients likely developed cross-reactivity against both platelet and neurogenic glycoproteins (the linking antigen) following predisposing infection. Plasmapheresis is a safe and equally effective choice of treatment incase IVIg or PE is not available or affordable in resource limited settings.

## Provenance and peer review

Not commissioned, externally peer-reviewed.

## Please state any conflicts of interest

Authors declare they have no personal or furnish relationship with any organization or people that would bias work.

## Please state any sources of funding for your research

No funding from any source.

## Ethical approval

Consent was taken from patient.

## Consent

Written informed consent was obtained from the patient for publication of this case report and accompanying images. A copy of the written consent is available for review by the Editor-in-Chief of this journal on request”.

## Author contribution

JK performed data collection, TH, JK, IQ, AK, and SJS performed literature search, drafted the manuscript, reviewed and approved the manuscript.

## Registration of research studies

1. Name of the registry: N/A.

2. Unique Identifying number or registration ID: N/A.

3. Hyperlink to your specific registration (must be publicly accessible and will be checked): N/A.

## Guarantor

Taimoor hussain and Jasvindar Kumar.
